# Association of loneliness with suicide risk and depression in individuals with schizophrenia: moderating effects of self-esteem and perceived support from families and friends

**DOI:** 10.1038/s41537-023-00368-7

**Published:** 2023-07-05

**Authors:** Yi-Lung Chen, Cian-Ruei Jian, Yu-Ping Chang, Shu-Ru Chao, Cheng-Fang Yen

**Affiliations:** 1 Department of Psychology, Asia University, Taichung, Taiwan; 2 Department of Healthcare Administration, Asia University, Taichung, Taiwan; 3 Department of Psychiatry, Kaohsiung Medical University Hospital, Kaohsiung, Taiwan; 4 Department of Psychiatry, School of Medicine, College of Medicine, Kaohsiung Medical University, Kaohsiung, Taiwan; 5 School of Nursing, The State University of New York, University at Buffalo, Buffalo, NY USA; 6 Department of Social Work, National Pingtung University of Science and Technology, Pingtung, Taiwan; 7 College of Professional Studies, National Pingtung University of Science and Technology, Pingtung, Taiwan

**Keywords:** Schizophrenia, Human behaviour

## Abstract

Loneliness is prevalent among individuals with mental illnesses. This cross-sectional survey study examined the moderating effects of self-esteem and perceived support from families and friends on the association of loneliness with suicide risk and depression in individuals with schizophrenia. In total, 300 participants (267 with schizophrenia and 33 with schizoaffective disorder) completed the University of California, Los Angeles, Loneliness Scale (Version 3); suicide module of the Mini International Neuropsychiatric Interview; Center for Epidemiologic Studies Depression Scale; Family and Friend Adaptability, Partnership, Growth, Affection, and Resolve Index; and Rosenberg Self-Esteem Scale. Moderation analysis was performed to examine the moderating effects of self-esteem and perceived support from families and friends on the association of loneliness with suicide risk and depression. The results found that self-esteem was significantly associated with a reduced magnitude of depression in participants with loneliness. In addition, perceived support from friends was significantly associated with a reduced magnitude of suicide risk in participants with loneliness. Our findings indicate the importance of intervention programs that strengthen support from friends and self-esteem in reducing suicide risk and depression among lonely individuals with schizophrenia.

## Introduction

Suicide and depression are prevalent among individuals with schizophrenia. A meta-analysis and systematic review revealed that the lifetime prevalence of suicidal ideation in individuals with schizophrenia was 34.5%^1^, much higher than that in the general population worldwide (9.2%)^2^. More than 50% of individuals with schizophrenia experience depressive symptoms in their lifetime^3,4^. Depression can increase the risks of relapse, violence, suicide, and substance-related problems and can worsen the quality of life and family relationships^3^. Early detection and intervention are crucial to preventing the serious consequences associated with suicide risk and depression in individuals with schizophrenia.

Factors that increase the risk of suicide and depression must be identified to develop intervention programs for individuals with schizophrenia. According to the diathesis–stress model of suicidal behavior^5^, interpersonal–psychological theory of suicide (IPTS)^6^, and three-step theory of suicide (TSTS)^7^, feelings of social isolation and alienation can increase the risk of suicidal ideation. Loneliness refers to an individual’s subjectively perceived distance between anticipated and actual levels of social connectivity^8^. Individuals with schizophrenia experience higher levels of loneliness than those without schizophrenia^9–12^. Loneliness is significantly associated with increased risk of suicide^13^ and depression^9,13,14^ in individuals with schizophrenia. These results indicate that loneliness should be considered an intervention target in suicide prevention programs for individuals with schizophrenia.

In other studies, age^15^ and sex^16^ have moderated the association between loneliness and suicide in the general population. Identifying the modifiable moderators of the association of loneliness with suicide risk and depression can improve develop client-specific and effective intervention programs. According to ecological systems theory^17^, reduced social support and self-esteem further strengthen the association of loneliness with suicide risk and depression. In other studies, both decreased social support^18–21^ and self-esteem^22^ have strengthened the association of loneliness with suicide and depression in older adults. However, no study has examined the moderating effects of global self-esteem and perceived support from families and friends on the association between loneliness and suicide risk among individuals with schizophrenia. Studies have usually integrated support from families and friends into the concept of social support^23,24^. Although poor family relationships have been identified as a risk factor for suicide among individuals with schizophrenia^25^, support from friends may have unique effects on the mental health of individuals with schizophrenia^26^, including a lowered risk of suicide^27^. By contrast, low self-esteem is significantly associated with loneliness^28,29^ and suicide^30^ in individuals with schizophrenia.

This cross-sectional study examined the moderating effects of self-esteem (an individual factor) and perceived support from families and friends (social factors) on the association of loneliness with suicide risk and depression in individuals with schizophrenia. We hypothesized that the association between loneliness and suicide risk would be weaker among individuals with schizophrenia with higher levels of perceived support from families and friends and higher self-esteem than among those with lower levels of perceived support and lower self-esteem.

## Methods

### Participants

Individuals diagnosed as having schizophrenia or schizoaffective disorder in accordance with the Diagnostic and Statistical Manual of Mental Disorders: Fifth Edition (DSM-5)^31^ 2013 were recruited from the psychiatric outpatient department of a university hospital and two community psychiatric rehabilitation institutes in Kaohsiung, Taiwan, from February 2022 to May 2022. The psychiatrists confirmed the diagnosis during a screening visit based on the results of clinical interview and chart reviewing. The participants were aged between 20 and 70 years. Individuals with any intellectual disability, substance use disorder other than nicotine use disorder, or cognitive dysfunction due to severe physical diseases (i.e., brain injury, cerebrovascular diseases, hepatic encephalopathy, renal failure) that would prevent them from understanding the aim of this study or participating in the interview were excluded. Eight psychiatrists confirmed the eligibility of 362 individuals and then invited them to participate in this study. In total, 300 (82.9%) individuals (139 men and 161 women) agreed to participate in the study. According to Green^32^, a study using general linear analysis models needs 50 + 8 × (the number of total independent variable) participants at least. The present study contained 18 independent variables; therefore, 300 participants were sufficient for statistical analysis. After obtaining written informed consent from the participants, trained research assistants conducted face-to-face interviews with the participants in interview rooms. During the interviews, the participants’ sociodemographic characteristics, loneliness, suicide risk, perceived support from families and friends, and self-esteem were assessed using a research questionnaire. Each interview lasted 20–30 min, with the exact duration varying for each participant. All participants were assured that their responses would remain confidential.

### Measures

#### Loneliness

The 20-item Chinese version^33^ of the University of California, Los Angeles (UCLA), Loneliness Scale (Version 3)^34^ was used to assess the participants’ levels of loneliness. Each item was rated on a 4-point Likert-type scale from 1 (never) to 4 (always). Nine items were reverse coded to be in line with the remaining items. The total score ranged from 20 to 80, with a higher total score indicating a higher level of loneliness. In previous research, the Chinese versions of the UCLA Loneliness Scale (Version 3) exhibited favorable psychometric properties among individuals with schizophrenia or schizoaffective disorder^33^. Cronbach’s α in our study was 0.90.

### Depression

The 20-item Mandarin Chinese version^35^ of the Center for Epidemiologic Studies Depression Scale (MC-CES-D)^36^ was used to assess the frequency of depressive symptoms in the preceding month. Each item was rated on a 4-point Likert-type scale from 0 (rarely or none of the time) to 3 (most or all of the time). The total score ranged from 0 to 60, with a higher total score indicating more severe depression. The MC-CES-D has satisfactory internal consistency (Cronbach’s *α* = 0.90), 1-week test–retest reliability (intraclass correlation reliability = 0.93), congruent validity (area under the receiver operative characteristic curves for major depressive disorder = 0.88–0.90)^37^, and construct validity^38^. Cronbach’s *α* in our study was 0.91.

### Suicide risk

Five items in the suicide module of the Mini International Neuropsychiatric Interview^39^ were used to assess suicide risk among the participants in the preceding month in terms of thoughts of death, desire to self-harm, thoughts of suicide, plans for suicide, and suicide attempts. Participants answered “yes” or “no” for each item. The total number of items that received a “yes” indicated the severity of suicide risk. The total score ranged from 0 to 5. Cronbach’s *α* was 0.71.

### Perceived support from families and friends

The five-item Chinese version^40^ of the Family Adaptability, Partnership, Growth, Affection, and Resolve (APGAR) Index^41^ was used to assess the participants’ perceived support from families. The Family APGAR Index was adapted into a Friend APGAR Index to assess the participants’ perceived support from friends. The Friend APGAR Index has been used in a previous study to assess perceived support from friends^42^. Each item was rated on a 4-point Likert-type scale from 1 (never) to 4 (always). The total scores of the Family APGAR Index and Friend APGAR Index ranged from 5 to 20, with higher total scores indicating higher levels of perceived support from families and friends, respectively. Cronbach’s α values for the Family APGAR Index and Friend APGAR Index in our study were 0.94 and 0.93, respectively.

### Self-esteem

The 10-item Rosenberg Self-Esteem Scale (RSES)^43^ was used to assess the participants’ global self-esteem. Each item was rated on a 4-point Likert-type scale from 1 (strongly disagree) to 4 (strongly agree). A higher total score indicated a higher level of global self-esteem. The RSES has high reliability and construct validity. The total score ranged from 10 to 40. Cronbach’s *α* in our study was 0.84.

### Psychiatric symptoms

The 30-item Chinese version^44^ of the Positive and Negative Syndrome Scale (PANSS)^45^ was used to assess the severity of the participants’ psychiatric symptoms. Psychiatrists rated each item on a 7-point Likert-type scale from 1 (absent) to 7 (extreme). According to van der Gaag et al. ^46^, the PANSS contains five modules assessing positive symptoms, negative symptoms, disorganization, excitement, and emotional distress. The mean scores of the five modules were used to represent the severity of psychiatric symptoms. The mean score of each module ranged from 1 to 7, with a higher mean score indicating more severe psychiatric symptoms. Cronbach’s α for the five modules in our study ranged from 0.65 to 0.80. Given that emotional distress was highly associated with depression in this study, only positive symptoms, negative symptoms, disorganization, and excitement entered into linear regression analysis models to prevent a collinearity problem.

### Sociodemographic characteristics

The participants’ sex (0 = female; 1 = male), age, education level, monthly disposable income (“What is the amount of money you are free to spend each month?”), marital status (separated or divorced vs. married or cohabited), and occupation (unemployed vs. full-time or part-time job) were collected.

### Data analysis

Statistical Analysis Software (SAS) 9.4 (SAS Institute Inc., Cary, NC) was used to perform statistical analysis. Sociodemographic data (age, sex, education level, and monthly disposable income), psychiatric symptoms, loneliness, suicide risk, depression, perceived support from friends and friends, and self-esteem were analyzed using descriptive statistics. Skewness and kurtosis were assessed to determine the normality of the continuous variables. According to Kim^47^, when the sample size is ≤300, the criteria for normality are skewness and Pearson’s kurtosis values within ±3.29. Our preliminary analysis revealed that suicide risk was not normally distributed, with the skewness and kurtosis values being 2.90 and 10.12, respectively. Upon transforming the variable of suicide risk to the log scale, the skewness and kurtosis values were 1.74 and 2.11, respectively, indicating that normality was a reasonable assumption.

The first linear regression analysis model was used to assess the associations of the predictor (i.e., loneliness), covariates (i.e., sociodemographic data and psychiatric symptoms), and potential moderators (i.e., perceived support from families and friends and self-esteem) with depression. The second model was used to assess the associations of the predictor, covariates (i.e., sociodemographic data, psychiatric symptoms, and depression), and potential moderators with the suicide risk. The association was reported using regression coefficient, and the magnitude of regression coefficient indicates that one-unit increase in the predictor, an expected change of units in the outcome. Moderation analysis was performed using the PROCESS v4.0.0 macro, which is based on linear regression modeling and developed by Hayes for SAS. Additional interaction terms between the corresponding predictors and moderators were added in the moderation analysis. The magnitude of regression coefficient of interaction term indicates that incremental effect of the predictor on the outcome when one-unit increase in the moderator. The Johnson–Neyman technique was used to determine whether the conditional effects of loneliness on the log of depression and suicide risk differed at given values for moderators (self-esteem and perceived support from friends and friends) and to assess the statistical significance of the conditional effects within the measurement range of the moderators. Significant transition points in the moderation models indicated that the conditional effects of loneliness on the log of depression and suicide risk significantly differed for the given values of the moderators. A two-tailed *p* value of <0.05 indicated statistical significance.

## Results

Table 1 presents the descriptive data of sociodemographic characteristics, psychiatric symptoms, loneliness, depression, suicide risk, perceived support from families and friends, and self-esteem as frequencies and proportions or as means and standard deviations (SDs). The mean age of the participants was 45.9 years (SD = 11.7 years); 48 (16%) participants were married or cohabited; 89 (29.7%) participants had full-time or part-time job; and their mean duration of education was 13 years (SD = 2.6 years). The mean monthly disposable income was NT$8230.2 (SD = NT$8366.6). The mean (SD) score for loneliness was 43.4 (11.1), indicating a moderate severity^48,49^. The mean (SD) score for depression was 16.6 (10.8), indicating a mild severity. The mean (SD) score for suicide risk was 0.4 (0.9), indicating a very mild severity. The mean positive symptoms, negative symptoms and disorganization scores on the PANSS ranged from 2.5 to 3.6 (SD = 0.9), indicating a mild to moderate severity; the mean excitement score was 2.6 (SD = 0.9), indicating an extremely mild to mild severity. The mean (SD) scores for perceived support from families and friends were 15.7 (3.6) and 13.3 (4.4), respectively, indicating moderately high levels. The mean (SD) score for self-esteem was 28.1 (5.5), indicating a moderately level.

**Table 1:** Participant characteristics (*N* = 300).

Variable		
	*n* (%)	
Gender		
Male	139 (46.3%)	
Female	161 (53.7%)	
Marital status		
Separated or divorced	252 (84)	
Married or cohabited	48 (16)	
Occupation		
Unemployed	211 (70.3)	
Full-time or part-time job	89 (29.7)	

	Mean (SD)	Range
Age (year)	45.9 (11.7)	20–70
Education (year)	13.0 (2.6)	6–20
Money that could be spent freely (NT dollar)	8230.2 (8366.6)	0-60000
Positive symptoms on the PANSS	3.5 (0.9)	2–6
Negative symptoms on the PANSS	3.6 (0.9)	1–6
Disorganization on the PANSS	3.6 (0.9)	1–6
Excitement on the PANSS	2.6 (0.9)	1–5
Loneliness	43.4 (11.1)	20–76
Perceived support from families	15.7 (3.6)	5–20
Perceived support from friends	13.3 (4.4)	5–20
Self-esteem	28.1 (5.5)	11–40
Depression	16.6 (10.8)	0–54
Suicide risk	0.4 (0.9)	0–4

*PANSS* Positive and Negative Syndrome Scale.

**Fig. 1:**
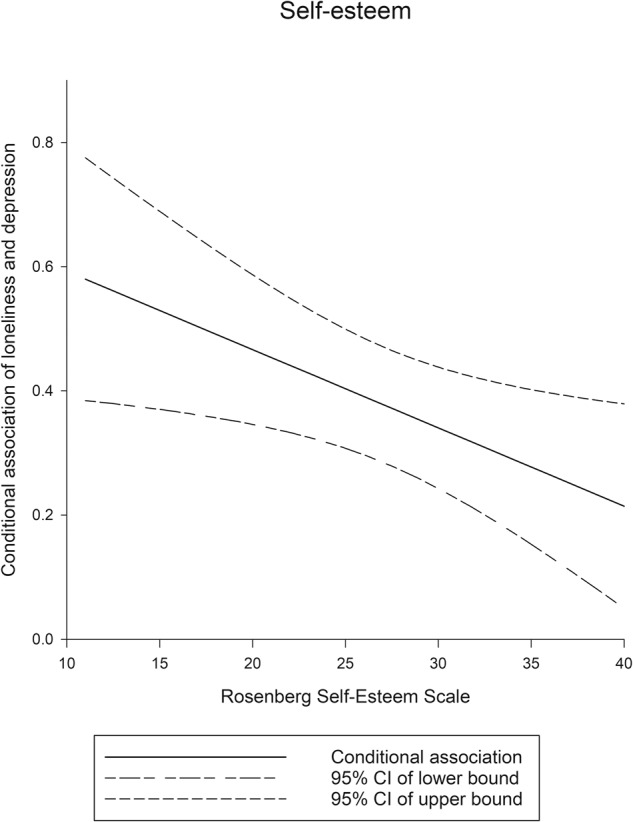
Moderating role of self-esteem between loneliness and depression.

A linear regression analysis model was used to examine the factors related to depression (Table 2). Loneliness and psychiatric symptoms were positively associated with depression, whereas self-esteem was negatively associated with depression. Moderation analysis was also performed to assess the possible moderating effects of self-esteem and perceived support from families and friends on the association between loneliness and depression. The results indicated that self-esteem moderated this association. Self-esteem was significantly associated with a reduced magnitude of depression in participants with loneliness (coefficient = −0.013, *p* < 0.01). The moderating effects are presented in Fig. 1. A higher level of self-esteem corresponded to a lower intensity of depression in participants with loneliness. Significant transition points were not observed on the basis of the Johnson–Neyman technique, indicating that a significant positive association remained throughout the range of the RSES for self-esteem (i.e., 10–40).

**Table 2:** Moderation effect of perceived support from families and friends and self-esteem on the association between loneliness and depression among individuals with schizophrenia.

	Unadjusted model	Moderation model		
		Perceived family support	Perceived friend support	Self-esteem
	*B* (se)	*B* (se)	*B* (se)	*B* (se)
Loneliness	0.415 (0.055)***	0.994 (0.184)	0.770 (0.123)***	0.719 (0.154)**
Gender	−1.043 (0.848)	−1.745 (0.941)	−1.999 (0.954)	−1.192 (0.830)
Age	−0.054 (0.039)	−0.071 (0.044)	−0.070 (0.044)	−0.045 (0.039)
Education	0.040 (0.176)	0.029 (0.197)	0.040 (0.199)	0.083 (0.173)
Money that could be spent freely^a^	0.122 (0.054)*	0.144 (0.061)	0.136 (0.061)	0.116 (0.054)
Marital status	0.284 (1.195)	0.606 (1.345)	0.859 (1.353)	0.346 (1.183)
Occupation	−1.144 (0.999)	−1.914 (1.120)	−1.877 (1.131)	−1.230 (0.987)
Positive symptoms	1.560 (0.569)*	1.151 (0.641)	1.189 (0.644)	1.365 (0.569)
Negative symptoms	−1.051 (0.581)	−0.990 (0.655)	−0.895 (0.663)	−0.940 (0.579)
Disorganization	2.486 (3.300)	1.811 (3.729)	1.999 (3.741)	2.193 (3.277)
Excitement	−1.708 (3.426)	−0.877 (3.875)	−1.325 (3.887)	−1.508 (3.405)
Perceived family support	0.216 (0.130)	1.115 (0.529)*	–	–
Perceived friend support	0.047 (0.113)	–	0.389 (0.386)	–
Self-esteem	−0.869 (0.095)***	–	–	−0.327 (0.248)
Moderation-term	–	–	–	–
Loneliness by perceived family support	–	−0.020 (0.0113)	–	–
Loneliness by perceived friend support	–	–	−0.011 (0.0087)	–
Loneliness by self-esteem	–	–	–	−0.013 (0.0054)*

Loneliness was measured by the UCLA Loneliness Scale (Version 3).

Depression was measured by the Center for Epidemiologic Studies Depression Scale.

^a^Analysis is presented as 1000 NT dollars per unit.

^*^p < 0.05, **p < 0.01, ***p < 0.001.

**Fig. 2:**
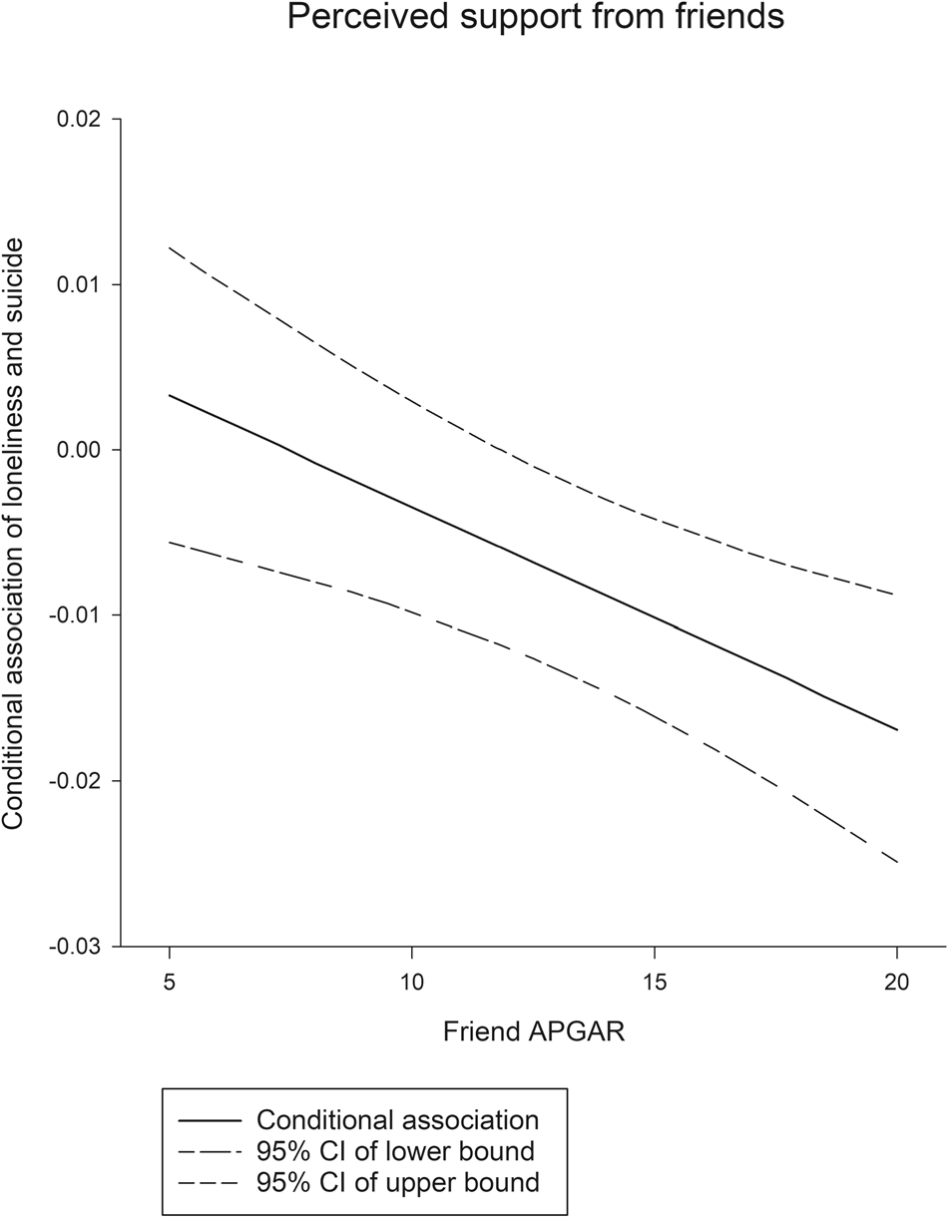
Moderating role of perceived support from friends between loneliness and suicide.

A linear regression analysis model was used to examine the factors related to suicide risk (Table 3). The results indicate that loneliness and depression were positively associated with suicide risk. Moderation analysis was performed to assess the possible moderating effects of self-esteem and perceived support from families and friends on the association between loneliness and log of suicide risk. The results revealed that after the effects of sociodemographic, psychiatric symptoms, and depression were controlled, perceived support from friends moderated this association between loneliness and suicide risk. Perceived support from friends was significantly associated with a reduced magnitude of suicide risk in participants with loneliness (coefficient = −0.001, *p* < 0.001). The moderating effects are presented in Fig. 2. Higher levels of perceived support from friends corresponded to a lower suicide risk among participants with loneliness. The Johnson–Neyman technique revealed that the relationship between loneliness and suicide risk was nonsignificant when the Friend APGAR Index score for perceive friend support was ≥12.

**Table 3:** Moderation effect of perceived support from families and friends and self-esteem on the association between loneliness and suicide risk among individuals with schizophrenia.

Variable	Unadjusted model	Moderation model		
		Perceived family support	Perceived friend support	Self-esteem
	*B* (se)	*B* (se)	*B* (se)	*B* (se)
Loneliness	0.010 (0.003)**	−0.002 (0.009)	0.010 (0.006)	0.002 (0.009)
Gender	0.016 (0.047)	0.026 (0.046)	0.024 (0.046)	0.018 (0.014)
Age	−0.003 (0.002)	−0.003 (0.002)	−0.003 (0.002)	0.000 (0.000)
Education	−0.007 (0.010)	−0.007 (0.010)	−0.010 (0.010)	0.024 (0.046)
Money that could be spent freely ^a^	−0.002 (0.003)	−0.002 (0.003)	−0.001 (0.003)	−0.003 (0.002)
Marital status	−0.082 (0.066)	−0.090 (0.065)	−0.092 (0.065)	−0.010 (0.010)
Occupation	−0.071 (0.055)	−0.071 (0.055)	−0.043 (0.054)	−0.001 (0.003)
Positive symptoms	0.016 (0.032)	0.018 (0.031)	0.022 (0.031)	−0.094 (0.066)
Negative symptoms	−0.005 (0.032)	−0.007 (0.032)	0.003 (0.032)	−0.064 (0.055)
Disorganization	0.057 (0.181)	0.054 (0.181)	0.076 (0.179)	0.016 (0.032)
Excitement	−0.061 (0.188)	−0.052 (0.188)	−0.074 (0.186)	−0.004 (0.032)
Depression	0.024 (0.003)***	0.023 (0.003)***	0.022 (0.003)***	0.074 (0.182)***
Perceived family support	−0.015 (0.007)	0.007 (0.0259)	–	–
Perceived friend support	−0.004 (0.006)		0.053 (0.0185)	–
Self-esteem	0.006 (0.006)	–	–	−0.065 (0.1889)
Moderation-term	–	–	–	–
Loneliness by perceived family support	–	−0.001 (0.0006)	–	–
Loneliness by perceived friend support	–	–	−0.001 (0.0004)**	–
Loneliness by self-esteem	–	–	–	0.023 (0.0033)

Loneliness was measured by the UCLA Loneliness Scale (Version 3).

Suicidal risk was measured by the suicide module of the Mini International Neuropsychiatric Interview.

Depression was measured by the Center for Epidemiologic Studies Depression Scale.

^a^Analysis is presented as 1000 NT dollars per unit.

*p < 0.05, **p < 0.01, ***p < 0.001.

## Discussion

Using unadjusted linear regression analysis models, this study revealed that loneliness, perceived support from families and friends, and self-esteem were significantly associated with suicide risk and depression among individuals with schizophrenia. Self-esteem was significantly associated with a reduced magnitude of depression among lonely individuals with schizophrenia. Perceived support from friends was significantly associated with a reduced magnitude of suicide risk among lonely individuals with schizophrenia. This study is the first to support the moderating effects of global self-esteem and perceived support from friends on the association of loneliness with depression and suicide risk among individuals with schizophrenia. The results indicate that in addition to reducing loneliness, intervention programs should increase self-esteem and friend support to reduce suicide risk and depression among lonely individuals with schizophrenia.

According to the IPTS^6^ and TSTS^7^, loneliness is a type of interpersonal and psychological pain that increases suicide risk. If loneliness persists or worsens, individuals may lose their sense of connectedness to people, which provides a sense of meaning. Consequently, suicide risk will be exacerbated. The TSTS indicates that social and individual factors may influence suicide risk^50^ and that friend support may buffer the harmful effects of loneliness by providing individuals with emotional and interpersonal connectedness and may further weaken the association between loneliness and suicide risk by balancing the pain caused by loneliness and expectations.

We discovered that self-esteem was significantly associated with a reduced magnitude of depression in lonely individuals with schizophrenia. Individuals with adequate self-esteem may adopt problem-focused coping strategies and not avoidant coping strategies when they encounter stress^51^. Adequate self-esteem may also reduce the tendency to distort social information and perceive unreal hostility^52^. Thus, depression related to loneliness may be less severe among individuals with adequate self-esteem.

As in in several Asian countries that attach great importance to familism and collectivism consistent with Confucian cultural values^53^, family support is highly valued among people in Taiwan. Family members often serve as primary caregivers and provide their loved ones with schizophrenia with emotional and practical support. However, we did not observe any moderating effect of family support on the association of loneliness with depression and suicide risk. While this result must be replicated and confirmed by further research, the result indicated that mental health of individuals with schizophrenia might be influenced by multiple individual and environmental factors but not only family support. Poor family relationships remain a risk factor for suicide among individuals with schizophrenia^25^ and thus warrant intervention.

### Implications

We offer several suggestions based on the results. First, loneliness should be routinely assessed for the early detection of depression and suicide risk among individuals with schizophrenia. Second, intervention programs designed to reduce loneliness in this population may contribute greatly to early identification and prevention efforts aimed to reduce suicide risk and depression. Several intervention programs for loneliness have been developed to improve social skills, enhance social support, create opportunities for social contact, and address maladaptive social cognition among individuals with loneliness^54^. A meta-analysis of randomized comparison studies revealed that the most successful interventions could address maladaptive social cognition^54^; however, the effects of such interventions on loneliness among individuals with schizophrenia have not been examined. Third, enhancing friend support is also recommended to reduce suicide risk among individuals with schizophrenia who experience loneliness. A meta-analysis study confirmed that social skill training can reduce the severities of negative symptoms and general psychopathology as well as enhance social cognition and performance in patients with schziophrenia^55^ and may help build social relationships with others. Since 2008, the Taiwanese government has been encouraging the establishment of community rehabilitation centers to help patients with schizophrenia to improve the ability of living independently, form peer groups, and enhance social support through group rehabilitation activities. Moreover, digital friend support intervention programs have been developed for individuals with psychotic disorders^28,56^. Fourth, self-esteem is an intervention target to reduce depression among individuals with schizophrenia who experience loneliness. Studies have reported that cognitive behavioral therapy^57^ and psychoeducation^58^ can increase the self-esteem of individuals with schizophrenia.

### Limitations

Some limitations of this study should be addressed. First, we collected data from the participants in the face-to-face interview conducted by research assistants. Although the method of collecting data could reduce missing data, single-rater and social desirability biases may have existed. Second, this cross-sectional study could not determine the temporal relationships and directionality of relationships among variables. Third, all the participants were recruited from psychiatric outpatient departments or community psychiatric rehabilitation institutes. Whether the results of this study can be generalized to individuals living in chronic psychiatric wards or those who do not visit psychiatric medical units for treatment remains unknown. Moreover, those with substance use disorders were excluded from this study; it might limit the generalization of the results of this study. Fourth, the psychometric propensities of the Friend APGAR Index used in individuals with schizophrenia warrant further examination.

## Conclusion

This study revealed the effects of loneliness on depression and suicide risk among individuals with schizophrenia. Loneliness warrants assessment and intervention to reduce depression and suicide risk among individuals with schizophrenia. Perceived support from friends and self-esteem are significantly associated with reduced magnitudes of suicide risk and depression among individuals with loneliness. Mental health professionals should implement intervention programs to enhance support from friends and self-esteem and reduce suicide risk and depression among lonely individuals with schizophrenia.

## Data Availability

The data that support the findings of this study are available from the corresponding author, upon reasonable request.
